# Building capability for clinician-led genomic change: insights from use and non-use of a theory-informed model for change

**DOI:** 10.3389/fgene.2025.1692703

**Published:** 2025-11-12

**Authors:** Melissa Martyn, Amy Clarke, Trang Do, Belinda Dawson-McClaren, Clara Gaff

**Affiliations:** 1 Melbourne Genomics Health Alliance, Parkville, VIC, Australia; 2 Murdoch Children’s Research Institute, Parkville, VIC, Australia; 3 Department of Paediatrics, The University of Melbourne, Parkville, VIC, Australia

**Keywords:** genomics, behavior change, physician, coaching, qualitative, complex intervention, change

## Abstract

Genomics is relevant to the practice of diverse medical specialties. However, integrating genomics into these specialties requires complex interventions to promote adoption by clinicians and organizational (service and hospital) re-design. Clinicians wanting to “champion” use of genomics rarely have experience in leading complex interventions. A practical evidence- and theory-informed model for change (M4C) designed for clinicians could increase likelihood of success of clinician-led complex practice change interventions. No such model could be identified, so we drew on three existing frameworks which collectively met our selection criteria to develop one. A case study approach was chosen to understand use of the M4C by clinicians leading three clinical change projects across hospitals. Informed by the COM-B system, we provided a toolkit and expert coaching to support use of the M4C. Mixed-methods data was collected: toolkit access; review of expert team notes; evaluation reports; focus groups and interviews. Coaching in all aspects of the M4C was provided, with clinicians particularly valuing coaching in action research and community engagement. Despite theoretical alignment of our strategies to promote adoption of the M4C, only 5/37 team members accessed the toolkit. Although clinicians did not independently engage with the M4C, they did embrace the concepts within it through coaching, with the expert team noting behavior change e.g., in engagement, leading to changes in project implementation. Access to expert support embedded within organizational (hospital) structures is needed to support clinicians to lead the system re-design required to incorporate innovations like genomics in healthcare.

## 1 Introduction

Despite convincing evidence for the value of genomic testing in patient care [reviewed in Bagger et al. (2024)], implementation of genomics faces health system-wide, organizational and individual level barriers (Friedrich et al., 2024; Gaff et al., 2017; Stark et al., 2019; Zebrowski et al., 2019). Even when reimbursement for test costs is available, uptake can be slow (Mordaunt et al., 2023; Schilling et al., 2025). As the relevance of genomic tests for diagnosis and treatment increasingly extends beyond the “traditional” providers of these services (i.e., specialist clinical genetic or precision medicine services), concerted effort is required to shepherd their availability in mainstream clinical care and adoption by clinicians.

Knowledgeable clinicians interested in incorporating genomics into patient care can be critical to diffusion of genomic medicine into mainstream clinical care (Hamilton et al., 2014; Mackley et al., 2025; Martyn et al., 2021). However, the actions of champions alone are unlikely to be sufficient for incorporation of genomics in healthcare. Skilled leadership of complex interventions (Skivington et al., 2021) is required to support the change needed for individual clinician level adoption as well as the system re-design (clinical service, hospital) needed at an organizational level (Holt et al., 2007). However, clinicians rarely have formal training in leading complex change (McKimm and Till, 2015; Stoller, 2014) and complex intervention guidance (for e.g., (Hull et al., 2019; O’Cathain et al., 2019)), is designed for researchers not clinicians. Strategies to build the capability of clinicians to design and lead complex change are therefore needed.

### 1.1 The melbourne genomics health alliance

The vision of the Melbourne Genomics Health Alliance (“Alliance”), funded by the members and the State Government in Victoria, Australia, is the sustained, effective and equitable use of genomics in the Victorian public hospital system (Gaff et al., 2017). The 2021–2025 Alliance program focused on funding projects which support systems re-design across and within organizations. This included 11 projects that “road-test” strategies to support the use of genomics by a range of healthcare professionals in diverse, real-world clinical settings: three “Statewide projects” aimed to inform equitable use of testing across a health system; three “clinical change projects” aimed to create change within specific clinical services across multiple independent organisations; and five ‘implementation projects’ which focused on change in a clinical service within a single organization.

A practical, evidence- and theory-informed model to guide action, termed the model for change (M4C), was used across these projects to support a consistent approach to their design and delivery. As the Alliance program has an explicit focus on building the capability of those within the system to lead the change needed to integrate genomics into the Victorian health system, the M4C was designed to be usable by clinicians, as well as by the expert Alliance team. To provide in-depth, real-life insights into how to support clinician-led efforts to integrate genomics into clinical care, clinicians in three projects were actively supported to use the M4C. Here we report our insights into the successes and failures of our approach.

## 2 Methods

The Melbourne Genomics program follows a pragmatic paradigm, focusing on identifying “what works” to support the change needed to incorporate genomics in healthcare (Weaver, 2018). A case study approach was chosen to understand use of the M4C by clinicians, as it allows in-depth, multi-faceted explorations of complex issues in their real-life settings to provide practical insights (Crowe et al., 2011), in keeping with a pragmatic paradigm.

### 2.1 Study setting

This study was set in the state of Victoria, Australia. Healthcare in Australia is provided by both the public and private sectors. Public hospital systems are the responsibility of each State/Territory and receive a mixture of State/Territory and federal government funding for different activities. In the private sector, some of the cost of consultations and procedures/investigations is reimbursed through the federal government funded Medicare Benefits Schedule (MBS). Clinicians working within the funded projects were employed by public hospitals; some projects also interfaced with private outpatient services.

The three “clinical change projects” were identified as most suitable for case study. These projects each targeted a different clinical service: nephrology, transplantation and memory loss clinics. Each was conducted at multiple hospitals, three which overlapped and they therefore shared common organisational (hospital) contexts. These three projects could thus provide rich data on the influence of the culture of the specialty and the culture of the hospital on application of the M4C. Each project team comprised a core leadership group of lead investigators and a coordinator (determined as n = 6, 11 and 7 for projects 1, 2 and 3 respectively) and a wider stakeholder team (determined as n = 9, 12, 7 for projects 1, 2 and 3 respectively). Genetic counselors (GCs) (n = 11) were employed as change agents on these projects and are included in the project membership numbers above. In addition to the M4C intervention describe here, GCs received other interventions which have been reported separately (Do et al., 2025; Do et al., 2024). The three projects were supported to use an action research approach, because of its suitability for providing insights into diffusion of innovation (Waterman et al., 2007).

### 2.2 Identification and development of a model for change

This study sought to use a common model to support a consistent approach to design and delivery across a range of clinical change projects. This was intended to enhance both the likelihood of success and the ability to synthesize insights across projects.

Firstly, a search was undertaken in October 2021 to identify existing theory- and evidence-informed models that could guide clinicians in the change leadership and processes required to undertake projects to integrate genomics in a particular clinical setting. Inclusion criteria were models that.

- Included components that reflected best practice in:
  - The change process
  - Research
  - Project governance and management
- Were developed using evidence-based methods
- Took a practical and pragmatic approach suitable for clinicians.

Publications not in English were excluded. The search string ((model OR framework OR guideline OR toolkit) AND (genomics OR genetics) AND (implementation) AND evidence-based) was applied in PubMed. Various combinations of terms were then used iteratively in PubMed and internet search engines. We also reviewed findings of a scoping review of structured approaches to implementation of clinical genomics (Brown et al., 2022). As no resources addressing all the selection criteria were identified, we progressed to develop a new model.

The second stage involved selecting and synthesising frameworks to inform the new M4C. Three frameworks addressing two of the selection criteria were identified in the initial search. A summary of the three frameworks used and the rationale for their inclusion in the M4C is shown in Table 1. Concepts pertaining to change processes, research and project governance were extracted from these and used to draft an initial high level diagrammatic change model. Face validity of this draft model was tested with two pragmatic samples: seven people with relevant expertise in at least one of the areas of education, evaluation, implementation science, research, project management, communications, clinical service delivery; potential users (3 clinicians). Structured feedback was sought using group (relevant expertise) and individual (clinician, relevant expertise) discussions. The initial draft was then refined to produce the M4C.

**TABLE 1:** The three frameworks informing the Model for Change (M4C), with rationale for inclusion.

	SEAchange (Harris et al., 2016)	Imp Res (Hull et al., 2019)	MRC (O’Cathain et al., 2019)
Purpose	To provide a pragmatic evidence-based guide to sustainable, effective and appropriate change in health services	To facilitate incorporation of implementation research core principes and concepts	To provide researchers with guidance on actions to take during complex intervention development
Target	Clinical teams	Researchers	Researchers
Setting	Clinical: Health service improvement	Applied health implementation research	Health service and public health research
Description	Guide outlines a model for evidence-based change based on a few simple rules	Ten implementation science consensus domains to inform implementation research design	Key principles and actions for consideration when developing interventions to improve health
Strengths	Focus on change processes, ease of application, pragmatic	Rigor of development, encourages use of implementation science theories, frameworks and models without being prescriptive	Comprehensive, rigor of development, logic model, dynamic
Limitations for use by clinicians	Single organization focus; Does not address project management and governance or research for change; no peer review	Requires some knowledge of implementation science. Doesn’t address change processes or project governance	Conceptual rather than practical; assumes knowledge of how to assess relevance and importance of suggested actions
Key contribution to M4C	Four steps in change process; non-linearity and agile approach	Consistent systematic approach to design promotes thinking about factors that hinder or facilitate implementation, including across contexts; clarity and consistent use of terms with definitions	Developmental thinking and refinement evaluation, application to multiple organizations

### 2.3 Promoting adoption of the M4C

**FIGURE 1:**
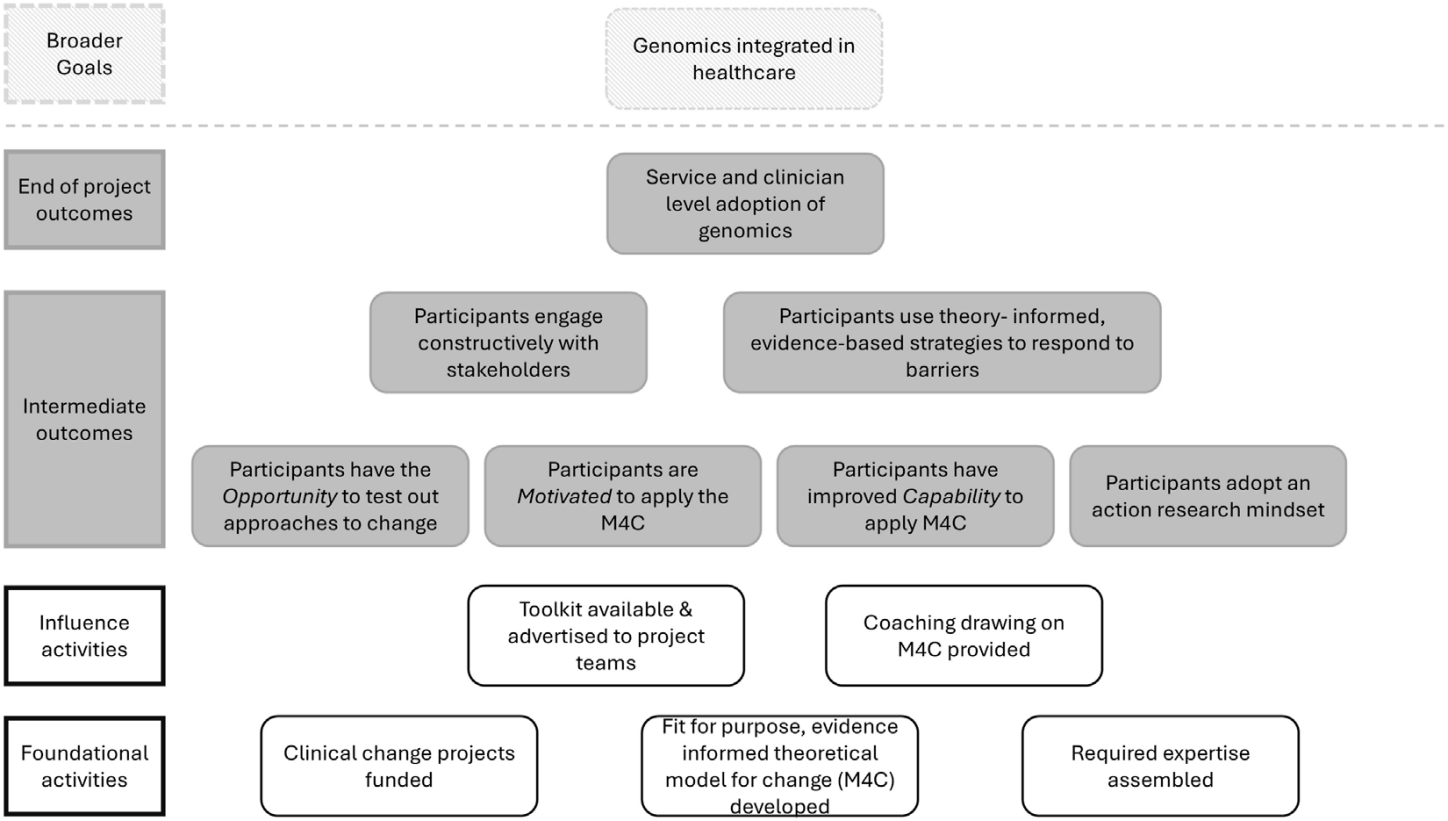
Program logic illustrating our program of work to support clinicians in the clinical change projects to use a model for change (M4C) to increase likelihood of successful service and clinician level adoption of genomics.

Our approach to supporting clinicians to use the M4C drew on the well-established COM-B model (Michie et al., 2011). That is, we understood that to use the M4C, clinicians would need to adopt behaviors to apply the M4C in their work to integrate genomics into clinical practice. As funded projects provide an *opportunity* for the new behavior of applying a M4C, developing clinicians’ *capability* and *motivation* to use the M4C should result in its application and increase the likelihood of integration of genomics in clinical services. The program logic outlining our approach is shown in Figure 1.

To influence adoption of the M4C by clinicians in the clinical change projects, two broad sets of activities were undertaken (see ‘influence activities’ in Figure 1; Table 2 for details). Influence activities were undertaken at cross-project assemblies that brought all projects together at key time-points for purposeful discussions to promote change (see Table 2 for details) and per project. Firstly, a toolkit (guide and videos) was prepared to assist clinicians to understand the M4C (See Supplementary Material S1). The M4C and toolkit were introduced via a presentation to project team members who attended the first assembly (n = 22). One project lead (from project 2) (n = 1), who could not attend the first assembly, subsequently received a one-on-one introduction to the M4C and toolkit. Project coordinators (n = 3) and genetic counselor champions (n = 11) were introduced to the M4C and toolkit when they commenced in their roles. Secondly, coaching based on the M4C was provided to increase motivation and capability to apply the M4C. Coaching was provided by members of an expert team, comprising expertise in community engagement, change management, action research, implementation science, project management, evaluation, advocacy and communications. Coaching was provided as part of the three cross-project assemblies, during tailored interactions in response to emergent needs identified by the expert team or project team members themselves, and *ad hoc* through regular meetings with project teams.

**TABLE 2:** Activities undertaken to influence adoption of M4C.

Activities	Description	N
Toolkit	*Written guide describing each element of the M4C, with links to further information* • 54 narrated videos and linked resources (median length 4 mins 53 sec, IQR 2’29”-8’53”)	Users: 5 users (GCs & coordinators) 1 user (coordinator) accessed 23 videos
Assemblies	*Three structured multi-project workshops*	Attendance:
	1. November 2022 Focus: initiation Introduction to M4C; focus on action research; community engagement and program theory & evaluation	22 project team members 1 hospital leader 2 community representatives
	2. March 2023 Focus: evaluation Project updates; Knowledge outputs; Evaluation & translation	28 project team members
	3. November 2024 Focus: sustainment Decision maker perspectives; crafting a value narrative	21 project team members 4 hospital executives
Coaching	*Single project capability building primarily to core team members*	Sessions N = 27
	Leadership	5 sessions (3 meetings with 1 project lead, one each with each of the others)
	Program evaluation	13 sessions 4x Background advice 2 meetings with 1 project, 1 with the others 3x Theory of change drafting - 1 per project 3x Introduction to program evaluation for coordinators - 1 per project 3x Revise theory of change – 1 per project
	Community engagement	7 sessions (2 with 2 projects, 3 with one)
	Project management & engagement	2 sessions

### 2.4 Data collection

Mixed-methods data collection techniques were used to explore use of the M4C by the project teams. The M4C toolkit (guide and videos) was hosted on a Sharepoint site to allow capture of utilization metrics (who accessed which files). Evaluation reports prepared following each assembly, including responses to formative evaluation surveys, qualitative feedback from participants and summaries taken from unstructured notes made by Melbourne Genomics researchers at each assembly, were reviewed by authors M.M and A.C. This was to identify data relevant to use of, or feedback on, the M4C. Members of the expert team kept unstructured records and notes of each formal coaching session provided outside the assemblies. A structured log was also kept to document situations arising with projects, actions taken and recommendations made, document impacts and identify emerging learnings. Authors M.M and A.C asked each member of the expert team to review their unstructured notes from each session and author A.C. reviewed the structured log to identify 1) the number and 2) the focus of each coaching session provided.

Qualitative data was collected from project leads and coordinators. Two focus groups with project coordinators were held after the introduction of the M4C (August 2023) and at the commencement of the evaluation phase (February 2024). The first covered use of the M4C and challenges they were encountering (to identify M4C concepts that would be relevant) and the second asked about how they were coordinating, supporting, documenting and reporting change. Project leads were interviewed after the projects had been completed (March-May 2025) to explore their recall and use of the M4C and views on coaching provided. Focus groups and interviews were audio-recorded, transcribed verbatim, and analysed using inductive content analysis (Vears and Gillam, 2022). Transcripts were read by the interviewer (T.D) who first selected data segments pertaining to use of the M4C and perceptions on coaching. These chunks of data were then coded for big-picture categories, verified by author M.M. Rather than assessing inter-rater reliability, authors M.M. and T.D. met frequently to discuss interpretation of the data to resolve discrepancies. Insights emerging from the qualitative data were discussed with the wider research team and with members of the expert coaching team to ensure consistency and validity.

## 3 Results

### 3.1 The model for change

**FIGURE 2:**
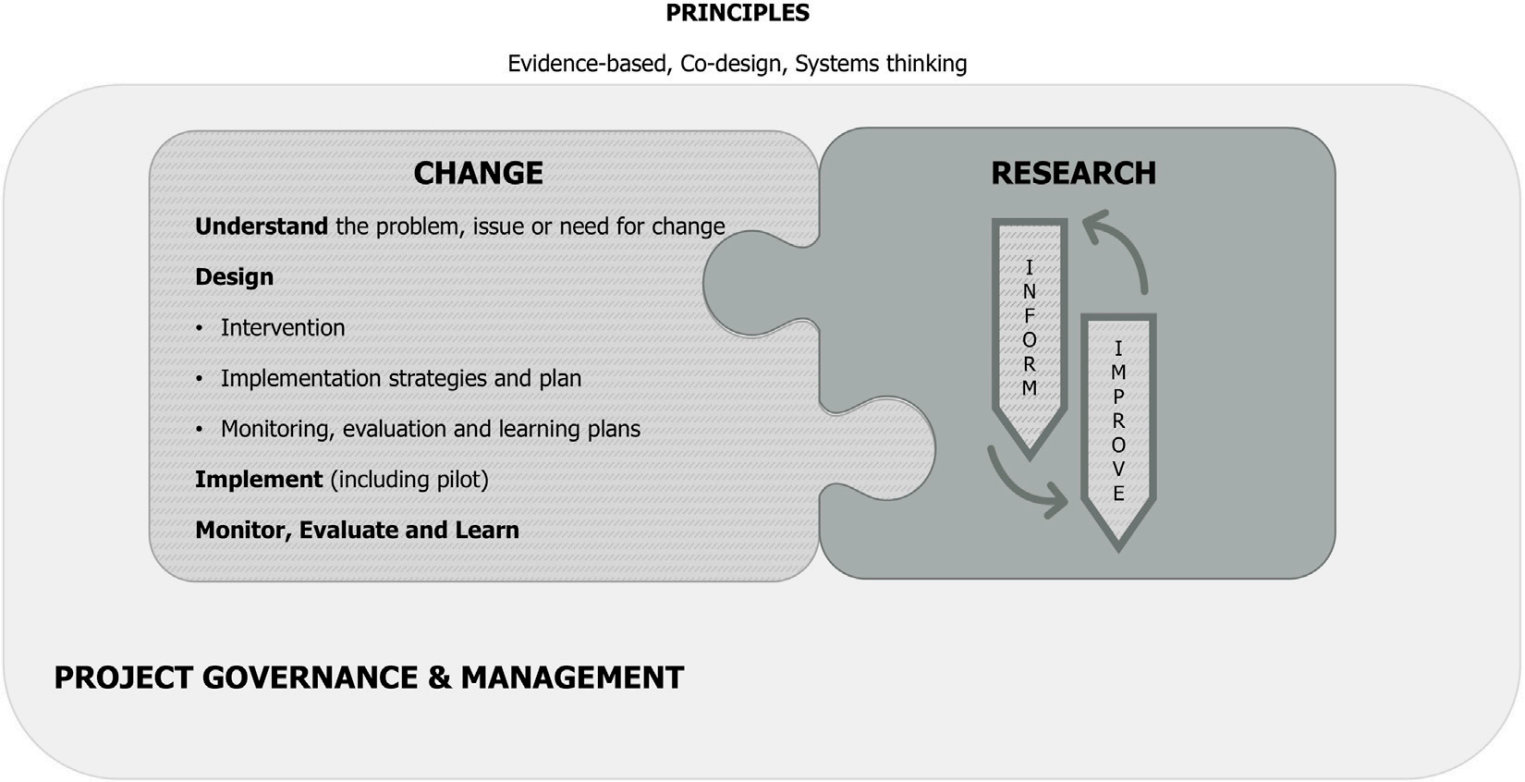
Iterated model for change.

The detailed M4C is shown in Supplementary Material S1. An iteration of the M4C made after conclusion of the case studies to reduce visual complexity is presented at Figure 2.

Three principles that enable effective, appropriate and sustainable change sit across the M4C: evidence-based, co-design and systems thinking. The importance of an evidence base and co-design are evident in all the frameworks that informed the M4C. “Systems thinking” extrapolates from the recognition of complexity by the ImpRes (Hull et al., 2019) and MRC (Skivington et al., 2021) frameworks and is intended to convey the need to think creatively, dynamically and respond to emergent issues in clinical change, rather than adopting a linear approach (Plsek and Greenhalgh 2001).

Change processes are presented in the hatched jigsaw piece. It's important to note that although the four subdomains (understand, design, implement, evaluate) are presented in a stepwise progression, the process is not usually linear–activities may be undertaken in parallel, or they may be iterative.

In parallel, developmental research (dark grey) offers the opportunity to both ‘inform’ and ‘improve’ change processes as they are occurring. This interrelationship is demonstrated by the interconnection of these jigsaw piece boxes.

The light grey outside box represents project governance and management, which supports both the change process and research activities.

### 3.2 Use of the M4C and toolkit

Of the 37 people to whom the M4C toolkit was explicitly promoted, it was accessed by five: three genetic counselors and two coordinators (Table 2). Only one of these, a coordinator, went on to review any of the videos. During the first focus group, this coordinator reported the videos assisted with understanding the coordination role in a change project, factors that might impact timelines, and being able to explain the purpose of the project set up (action research). One coordinator stated she lacked time to access the toolkit, with project tasks her priority. None of the three lead project investigators accessed the toolkit (guide or videos).

In post-project interviews, project leads did not report independently engaging with the M4C. All commented they had relied on others to engage with it and provide advice to ensure they incorporated relevant concepts into the project. They cited this responsibility as belonging to (variously): the expert team; a project team member with implementation science expertise; and/or the coordinator.

“I think [the expert team], they've all been involved in incorporating that model of [change] in our project … whatever it was recommended from [them], we've incorporated that. I'm assuming that was based on [the] model.” (project 1).

### 3.3 Coaching

Coaching on the M4C was provided to case study project teams, primarily to ‘core’ team members, but also to ‘wider’ team members through assemblies and project meetings. Coaching was provided in all three components of the M4C. As an example, one project encountered a challenge in getting genomic test reports to treating teams at the beginning of the project. The expert team prompted them to consider who they needed to engage with at the sites to understand how the treating team usually accessed results (stakeholder engagement) and to help them understand the needs of recipient teams (change process–understand the problem). This prompted project members to develop a new process to alert treating teams to result reports and furthermore to develop new summary communications tailored to using teams. This influenced ability of treating teams to use the genomic report information in patient care.

#### 3.3.1 Change processes

Articulating a theory of change and visually representing change pathways in a program logic was new to most project team members. With coaching, teams were able to shift to their focus from clinical outcomes to articulate change outcomes and link evaluation and reporting to their program logics, which were developed by the member of the expert team with expertise in program evaluation, in consultation with the teams:

“…whenever we had to think about results or outcomes, and say how are our outcomes matching up against the structure of the study … then we come back to that structure [the program logic]” (project 3).

A convincing value argument is a key factor in sustainability of pilot change projects (Martin et al., 2012). To provide coaching on this, the final assembly introduced teams to a framework, the ‘And But Therefore’ (ABT) framework (Olson, 2020) to help project team members articulate a value case for an aspect of their project. This was well received, with all 19 respondents to the evaluation survey strongly agreeing or agreeing the ABT framework was useful and would help them structure communication going forward. Qualitative feedback indicated this was a “key skill”:

“Learning how to pitch developments for the people who need to hear it, so pitching innovation, is the key skill that clinicians are missing…”

#### 3.3.2 Research

Action research was a new concept to most project team members, reflected in comments such as that of an attendee at the initial assembly who had “never heard of it”.

“That [action research conversation] was unbelievably amazing. I'd never really thought about it that way … It's not just doing research. It's about periodically stopping and taking notes. You know, taking stock of what you're doing… So it was very interesting how to actually formally capture what you're doing. And then, it's like in when you're running a race, you're just running but periodically stopping and looking back to see, you know, how far you've come.” (project 1).

Project team members had experience in clinical research, often in clinical trial research, but less experience of developmental research and evaluation. The idea of flexible rather than fixed implementation was novel. Coaching was provided to support this developmental mindset with project leads reflecting on the value of this approach for change projects:

“You’ve got to keep modifying as you go so you can actually be successful. That just means that our project has taken so many different shapes in so many different departments. Initially I was a bit skeptical about how is it going to work. There’s one model that’s being followed in one department, another model being followed in another department. But eventually, that was the actual success point. So my advice to anyone would be, be flexible, and keep getting constant feedback from the departments”. (project 1).

“I enjoyed very much the idea of the way these clinical change projects were developed from a very unformed, conceptual start, where the original pitches were brief and were focused on the quality of the ideas, rather than the detail. And then moving through that process of discussion and refinement … it allowed broad ranges of input into that design process. So we didn’t have to come with it completely tied down at the beginning.” (project 3).

#### 3.3.3 Project management and governance

Stakeholder engagement, particularly community engagement, were areas of the M4C the expert team provided significant coaching in. A community engagement coordinator supported teams to think about how community engagement might enhance their project and how community members could be meaningfully engaged. This was highly valued by project leads in the post-project interviews:

“…we had really good involvement from consumers … which I wouldn't have thought to do either because the usual model is you design your project, you do your ethics and everything and then you just inform consumers. But this was not how this was, so I really appreciate it all of that guidance … ” (project 1).

“I’ve had lots of projects which have involved consumers, but I don’t think we’ve ever had such a good interaction with a consumer group and had so much helpful commentary and thoughtful interaction. It really was superb. I thought [community engagement coordinator] did a great job at bringing that group together, and then facilitating the meetings.” (project 3).

### 3.4 Reported value of coaching

Project leads reflected that the expert coaching and support enhanced the impact of their projects and provided space for critical reflection:

“if you just left us to do just, hey, researchers, here's your funding and off you go, I don't think we would have achieved what we've achieved now because we don't really have the expertise, but also not that zoom out understanding” (project 1).

“It was really good to also have extra pairs of ears just listening to what's going on with the project and asking questions that we wouldn't have thought of asking ourselves.” (project 2).

Ongoing, there is a desire to have access to similar support “I think having a support group like this would be ideal.”

### 3.5 Observed changes in behavior

We observed all projects changed their intended models in response to stakeholder feedback and challenges encountered. Consumer engagement in two projects went well beyond the original scope, impacting project design and delivery. Action research approaches were embraced by the leads and project coordinators and adopted in all projects.

## 4 Discussion

We used a systematic approach to support design and delivery of a portfolio of clinical projects to engender the complex change needed to support adoption of genomics in healthcare. Informed by the COM-B system (Michie et al., 2011), we provided an *Opportunity* for clinicians in three clinical change projects to apply a practical, theory- and evidence-informed M4C. We undertook a range of activities to improve clinicians’ *Motivation* and *Capability* to apply a theory-informed approach to change, with mixed success. To provide insights that may assist others seeking to promote healthcare change, our discussion is structured around the program logic and the COM-B system.

Activities to *Motivate* clinicians to adopt and apply the M4C were informed by the intervention and policy support wheels of the COM-B system’s behavior change wheel (BCW). We raised awareness of the M4C (BCW intervention: education), introducing it at the first assembly, and emphasized its value for planning and implementing change (BCW intervention: persuasion). We also developed a guide and toolkit of short videos to improve clinicians’ capability to use the M4C (BCW intervention: training/education).

Even with the number, breadth and theoretical alignment of these activities to motivate project clinicians, independent engagement with the M4C was limited. The theoretical domains framework (Cane et al., 2012), which maps to the COM-B system, and diffusion of innovation in service organizations (Greenhalgh et al., 2004) suggest some factors that may explain this. Qualitative data suggested leads did not see engagement with the M4C as part of their professional role. Despite experiencing challenges whilst leading the change projects, this was not enough to create sufficient ‘tension for change’ i.e., to independently consult the M4C. Our presumption that project leaders and coordinators could be sufficiently motivated by the strategies we employed to consciously use the M4C to identify theory- and evidence-informed strategies to respond to barriers was not supported.

Experiencing challenges did motivate clinicians to seek support, and the multidisciplinary program team provided coaching to improve their *Capability* to draw on theory in their response. Experiential learning theory, specifically Kolb’s learning cycle (Yardley et al., 2012), posits that after a challenging concrete experience, space is required for learners to reflect and abstract learning from the experience and decide what they will do in response. Coaching can provide space for reflection, supporting experiential learning (BCW intervention: education). Professional learners generally face two types of challenges: technical and adaptive (Powell and Kusuma-Powell, 2015). Whilst technical challenges can be addressed with information, adaptive challenges require transformational learning. Coaching can assist with the critical examination and reframing required for transformational learning (Berta et al., 2015; Powell and Kusuma-Powell, 2015) (BCW intervention: modelling). Coaching enables professionals to draw not only on their existing knowledge and beliefs, but also on the expertise of the coach, theorized to improve success of the response to the barrier.

Coaching fostered a developmental research mindset and *Capability* to use action research skills. Despite low initial knowledge of action research, with coaching this was embraced by project teams. We observed that operational tensions acted as a barrier to developmental thinking, pushing teams towards a focus on ‘delivering’ projects rather than fostering change. For example, timeline pressures favored minimizing time spent in consultation about what processes to implement at different sites and encouraged standardization across sites, rather than the wide engagement and processes tailored to context that a change mindset would encourage. Change project management differs from traditional project management (Gordon and Pollack, 2018), requiring adaptive processes due to differences in contexts, particularly across organizations. Activities are not defined in advance by managers, rather stakeholder engagement is critical to achieve high level agreement on goals and navigate how best to achieve these in different contexts (Gordon and Pollack, 2018). In contrast, traditional project management involves clearly defining deliverables and delivering these to a schedule. We would advise others seeking to build capability for change to make these distinctions apparent and anticipate operational tensions that may tip teams towards delivery.

We did observe a shift in clinicians’ *Behavior* as they encountered challenges during the clinical change projects. To what extent this represents transformational learning, i.e., whether this will influence their change leadership behavior in the future, will only become apparent with time. Our multidisciplinary expert team encouraged reflection and focused on introducing concepts from theory of change and participatory action research, providing external capacity for transformational learning (Berta et al., 2015). There is evidence that coaching and action learning, such as that we provided, improves change leadership skills (Cleary et al., 2020; McNamara et al., 2014) and ability to undertake quality improvement (Giesen et al., 2024; Walunas et al., 2021), but evidence for long term impact is lacking.

Integration of genomics in health systems requires individual level (clinician adoption), organizational level (changes within hospitals) and wider system change (e.g., policy level). The Alliance program undertook work at multiple levels of change; here we have described only evaluation of collaborative projects led by clinicians to create change within clinical services. While, during the competitive process used to select these projects, it was emphasized that they would be part of a wider study on creating change, team members did not directly engage with the M4C itself. They did engage with - and reported finding useful - the concepts of the M4C through coaching and reported modifying their behavior in response.

To shift from programmatic coaching, such as that provided here, to spread and scale access to coaching to sustain change leadership capability is a challenge. At an organisational level, agreement on the value of such coaching and leadership support for a mechanism to enact it would be crucial for spread and scale up (Côté-Boileau et al., 2019). Embedded knowledge brokers (CHSRF, 2003) and communities of practice (Wenger, 2011) are two examples of mechanisms that can facilitate the actions of change makers (Do et al., 2025). Across organisations, academic health service partnerships (Robinson et al., 2020) could provide access to similar coaching expertise. There are no doubt other mechanisms that could replicate the function that coaching served here; the key will be to replicate the adaptive facilitation (Alagoz et al., 2018) using a mechanism that is fit for context (Papoutsi et al., 2024).

Limitations of this study include that we cannot know to what extent the M4C would be useful in the absence of an expert team able to provide coaching. Our intention was to give these clinicians a foundation–through the M4C–which they could draw on again in other contexts to engender change. We chose to introduce them to M4C concepts as they were salient, that is in ‘real time’ as they were working to make change and encountering challenges. However, we can only speculate about the impact this experiential learning will have on transforming their behaviors in the future. Others have explicitly trained participating clinicians in complex change leadership (Thakore, 2022) or in particular implementation science approaches (Taylor et al., 2020). Although the impact of the ‘Hawthorn effect’ on observational studies of behaviour change is uncertain (McCambridge et al., 2014), we cannot discount that those involved in the case study projects may have changed their behaviour because they knew they were being studied. Inherent in the choice of a case study approach, we acknowledge we studied only three projects and others may have used the M4C differently. Finally, some members of the research team held dual roles as coach and as researchers/evaluators; despite our efforts to encourage reflexivity, this may have introduced biases.

## 5 Conclusion

Our study underscores the importance of access to expert support, in this case to clinical change expertise. Of the approaches we tried with our projects teams to develop clinicians’ capability and motivation to use the M4C, coaching was the most utilised. The advantage of this approach is that it can be tailored to meet specific learner needs at any given moment. Toolkits and stand-alone resources lack this flexibility and–in our experience–are not perceived as relevant to those responsible for making change. Organizations (hospitals) and healthcare systems need to consider how they can incorporate access to such expertise into their structures to ensure clinician champions have the necessary skills in, and understand the importance of, complex change leadership.

## Data Availability

The datasets presented in this article are not readily available because the data supporting the conclusions of this article will be made available by the authors upon reasonable request. Requests to access the datasets should be directed to melissa.martyn@mcri.edu.au.
