# Supplementary material for: Building capability for clinician-led genomic change: insights from use and non-use of a theory-informed model for change
Source: Front Genet. 2025 Nov 12;16:1692703. doi: 10.3389/fgene.2025.1692703 (PMC12646539; doi:10.3389/fgene.2025.1692703)
Supplement: Supplementary file 1 [file Supplementaryfile1.docx]

Genomics in Practice Toolkit

A (shortcut) guide to effective, appropriate, and sustainable change


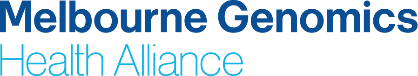


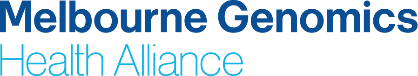
**Citations**

#### This document should be cited as:

Melbourne Genomics. Genomics in Practice Toolkit Guide to effective, appropriate, and sustainable change. Melbourne, Australia. 2022

#
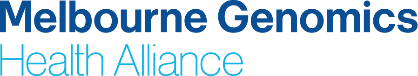
Genomics in Practice Toolkit

Introduction

The Genomics in Practice Toolkit, ‘the Toolkit’, provides resources to enable clinicians and other health service staff to design and deliver successful and sustainable change projects implementing genomics in real world clinical settings.

Audience

The Toolkit is aimed at health professionals, health service managers and scientists who want to introduce, increase uptake or enhance the use of genomics in clinical care, but find themselves in the common situation of having little knowledge or experience in implementing change and often limited time and resources for their projects.

Focus

The focus of the Toolkit is on ‘clinical change projects’ – initiatives undertaken by health service staff seeking to improve practice. It assumes that these are not research projects and that they are not conducted by researchers. The Toolkit provides practical assistance to project teams unfamiliar with the science of change or lacking experience in project governance and management, enabling them to achieve optimum outcomes within the time, knowledge, skills and resources available.

Components

This concise Toolkit outlines key components of the Model for Change, with learning objectives and links to the instructional video(s) for each topic.

A suite of videos outlines:

- Principles of best practice in designing and delivering projects implementing change
- Handy hints and tips for success
- Advice on how to anticipate and avoid common pitfalls

How to use the Toolkit

We recommend you **watch the videos in order** of this Toolkit, as information in earlier videos is built upon throughout. However, we acknowledge you will find some parts more useful at different stages. We recommend **watching the overview video and reading the learning objectives** for each topic so you have an understanding of which parts are most relevant to your work right now. The **videos** are broken into short segments, which will enable you to learn and refresh concepts quickly.

The **guide** follows the contents of the videos. It provides the links to each video segment, and the learning objectives for each topic.

Evaluation

There is a link to a brief survey at the end of each topic to help us evaluate the usefulness of the resources provided. Your feedback is appreciated.

#
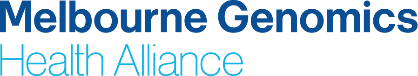
Model for Change

Introduction

Clinical change projects provide a great opportunity to learn about implementation of genomics. However, they also bring two significant challenges.

Projects will be undertaken by different teams, across different organisations, in different clinical settings, with different genomic applications. There is likely to be lack of consistency in project design and delivery, which may limit generalisability of findings.

Because clinical project teams have different levels of experience and expertise in the science of change or delivering complex projects there is a risk that they are not successful or sustainable.

These opportunities and challenges led Melbourne Genomics to develop a model for change that would meet the following aims.

1. To enable a **consistent**, **systematic** approach to the design and delivery of projects implementing clinical change based on the **principles of** **best practice**
2. To facilitate **testing and refinement** of interventions, implementation strategies, and evaluation plans for subsequent **replication or research**

Video

This video provides an overview of the aims, principles, components, outcomes and outputs of the model for change.

### Learning objective

To understand how the model for change will

- Increase the likelihood of successful and sustainable projects
- Contribute to the body of knowledge in implementation of genomics

### Watch the video

Toolkit overview

Model for Change Overview

**Click here to complete a survey to evaluate your learning.**

Model for change key messages Note: iterated model is presented in Figure 2, Martyn et. al. (2025)


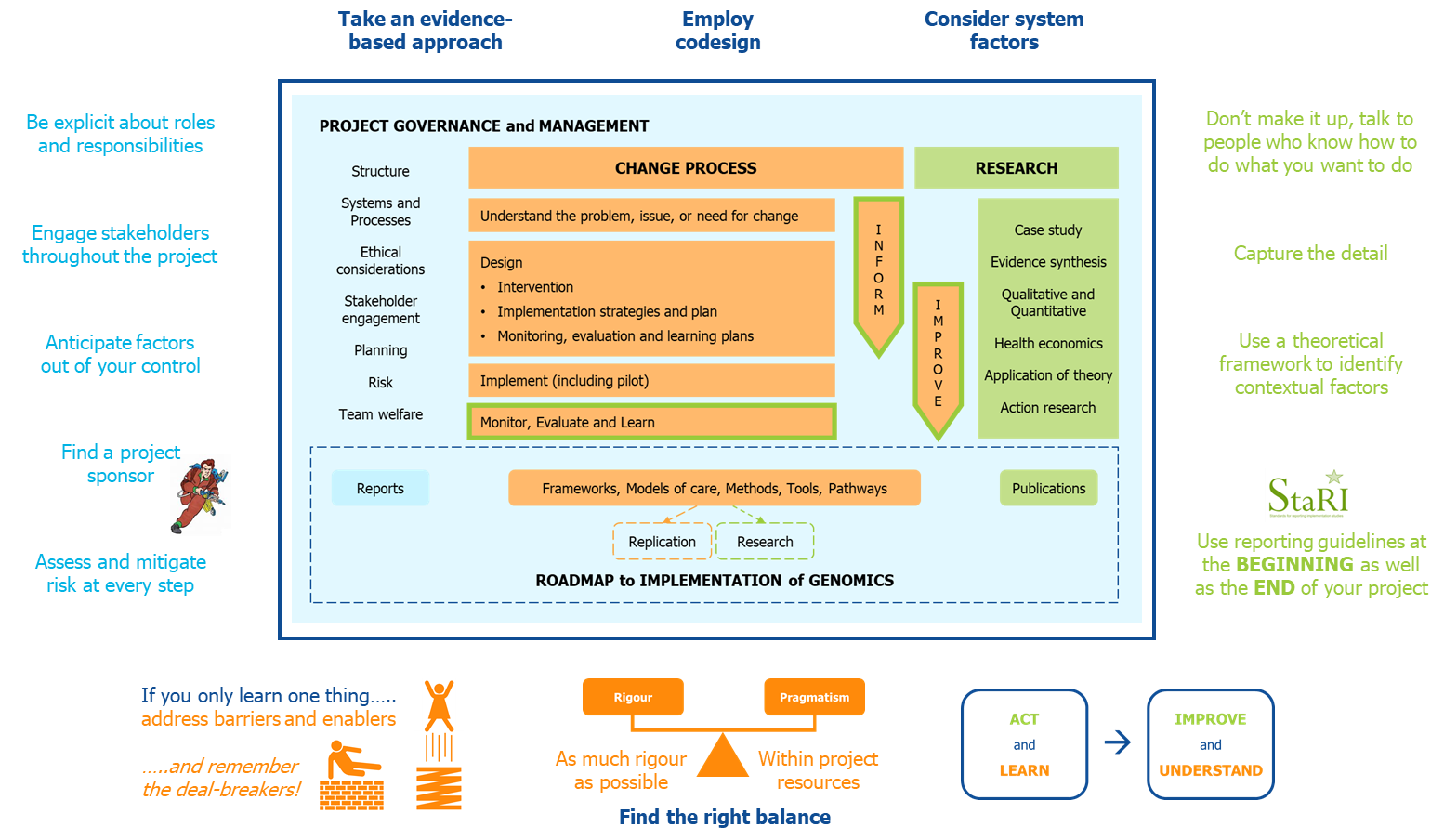


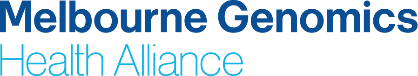
Principles

Introduction

To achieve effective, appropriate, and sustainable change, the model is underpinned by three principles.

**Effective** change is informed by **evidence**.

**Appropriate** change is informed by **codesign**.

**Sustainable** change is informed by **system factors**.

Videos

This module explores the three core principles and why they are needed to achieve effective, appropriate, and sustainable change.

### Learning objectives

- To understand each approach and how it underpins the model for change
- To appreciate the role and importance of each approach in achieving change
- To be able to apply each approach in clinical change projects

### Watch the videos

Principles module overview

Effective change

Appropriate change

Sustainable change

Principles module review and reflection

**Click here to complete a survey to evaluate your learning.**


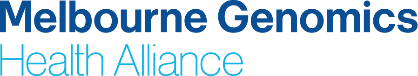

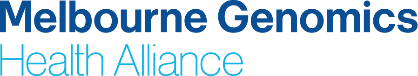
Project governance and management

Introduction

The model for change has three components. The first is project governance and management.

Videos

This module provides guidance on best practice in project governance and management and describes practical suggestions on how to avoid common errors across domains that include structure, systems and processes, ethical considerations, stakeholder engagement, planning, risk, team welfare and reporting.

### Learning objective

To understand, and be able to implement, the requirements of good project governance and management

### Watch the videos

Project governance and management module overview

Project structure: Relationships

Project structure: Roles and responsibilities

Systems and processes

Ethical considerations

Stakeholder engagement

Planning: Getting started

Planning: Timelines, milestones, deliverables

Planning: Factors that affect timelines

Risk

Team welfare

Reporting

Project governance and management recap and reflection

**Click here to complete a survey to evaluate your learning.**


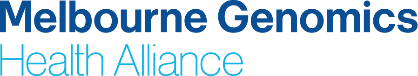
Change process

Introduction

The second component of the model for change is the change process itself. It includes:

- Understanding the problem, issue or need for change
- Designing the intervention, implementation strategies, and monitoring, evaluation and learning plans
- Implementing
- Evaluating
- Informing decisions and improving methods and materials
- Refining the outputs of the change process for use in other health settings and/or testing in research

Videos

This module provides guidance on best practice and offers practical suggestions to enhance the change process and avoid common errors.

### Learning objective

To understand, and be able to implement, the project activities in a process aiming to achieve change in a clinical setting

### Watch the videos

Change process module overview

Inform and improve

Understand the problem, issue, need for change

Design the intervention

Design the implementation strategies and plan - introduction

Design the implementation strategies and plan - barriers and enablers

Design the implementation strategies and plan - implementation strategies

Design the implementation strategies and plan - implementation plan

Design the monitoring, evaluation and learning plans - introduction

Design the monitoring, evaluation and learning plans – MEL framework

Design the monitoring, evaluation and learning plans – MEL plan

Implement

Monitor, evaluate and learn

Refine the outputs, recap and reflection

**Click here to complete a survey to evaluate your learning.**

#
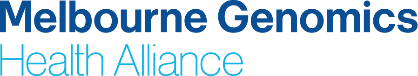
Research

Introduction

The focus of the Toolkit is on clinical change projects undertaken by clinicians, health service managers and scientists; not on research projects undertaken by researchers. However, research is still important in this context.

The role of research in clinical change projects is to inform, to improve, and to monitor, evaluate and learn.

Videos

This module includes the role of research in clinical change projects, a summary of key messages, and things to consider when publishing research findings.

Research governance and ethical considerations are included in the module on project governance and management.

### Learning objective

To understand where and how research fits into clinical change projects

### Watch the videos

Research module overview

Role of research

Key messages

Publishing

Research module recap and reflection

### Evaluation prompts

- Did you learn anything new?
- Did anything surprise you?
- How has this challenged what you were thinking about your project?

**Click here to complete a survey to evaluate your learning.**

#
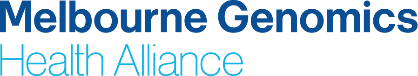
Action Research

Introduction

Action research plays an important role in delivery of successful and sustainable clinical change. It helps you to get over or around barriers you encounter during the project, to build on the enablers, to learn what works and doesn’t work, and to explain why things turned out the way they did at the end of the project.

Videos

This module defines action research and provides explanations of why we should do it, who is involved, when and how to do it, what tools are available and how to use them.

### Learning objectives

- To understand the role of action research in clinical change projects
- To understand the principles of action research
- To be able to apply action research methods

### Watch the videos

Action research module overview

What is action research?

Why do you do action research?

Who is involved in action research?

When do you do action research?

How do you do action research?

Which tools to use?

How do you pull it all together?

Action research recap and reflection

### Evaluation prompts

- Did you learn anything new?
- Did anything surprise you?
- How has this challenged what you were thinking about your project?

**Click here to complete a survey to evaluate your learning.**

#
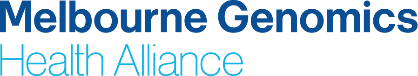
Complex interventions

Introduction

Interventions used to implement change in practice are usually considered to be ‘complex interventions’. As the name suggests, they add complexity to project design and delivery.

Videos

This module clarifies some confusing and inconsistent terminology, defines complex interventions and the implications they bring to clinical change projects, and explains the process of developing complex interventions and how this can enhance future research.

### Learning objective

To understand complex interventions and the implications for this complexity in clinical change projects

### Watch the videos

Complex interventions module overview

Terminology

Complex interventions

Implications for clinical change projects

Development of complex interventions

Complex interventions recap and reflection

### Evaluation prompts

- Did you learn anything new?
- Did anything surprise you?
- How has this challenged what you were thinking about your project?

**Click here to complete a survey to evaluate your learning.**
